# Improving the methodological quality of randomized clinical trials assessing psychotherapy for borderline personality disorder: Recommendations for the future

**DOI:** 10.3389/fpsyt.2022.1053844

**Published:** 2022-12-14

**Authors:** Sophie Juul

**Affiliations:** ^1^Copenhagen Trial Unit – Centre for Clinical Intervention Research, The Capital Region, Copenhagen University Hospital – Rigshospitalet, Copenhagen, Denmark; ^2^Stolpegaard Psychotherapy Centre, Mental Health Services in the Capital Region of Denmark, Copenhagen, Denmark

**Keywords:** personality pathology, borderline personality disorder, psychotherapy, clinical research methodology, evidence-based medicine, evidence-based health care

## Introduction

Borderline personality disorder (BPD) is a debilitating psychiatric disorder affecting 1–6% of the population (1, 2). BPD symptoms include identity diffusion, affective dysregulation, and often high rates of self-harming behavior and suicide-related mortality (3, 4). Today, psychotherapy is the most widely used intervention for treatment of BPD, and the efficacy of different specialized psychotherapies for BPD has already been assessed in many randomized clinical trials (5).

Evidence-based medicine is based on the fundamental principle that high-quality research should guide practice and decision-making in the care of individual patients (6). The systematic review of randomized clinical trials is considered the gold standard when estimating intervention effects (7). However, even at the top of the evidence hierarchy, methodological challenges may still occur in the design, conduct, analysis, interpretation, and publication of trial results. These challenges from individual trials may ultimately skew the results and conclusions drawn from systematic reviews of these trials.

A Cochrane review by Storebø et al. assessing the beneficial and harmful effects of all psychological therapies for BPD was published in 2020 (5). The authors concluded that dialectical behavioral therapy (DBT) and mentalization-based therapy (MBT) were more effective than treatment as usual on a number of patient-important outcomes (5). However, all results were based on low-quality evidence (5). The authors used the original Cochrane risk of bias tool to assess bias (8), and the Grades of Recommendations, Assessment, Development, and Evaluation (GRADE) guideline to assess the certainty of evidence (9).

Based on these methodological assessments (5) combined with my personal experiences with conducting a randomized clinical trial assessing psychotherapy for BPD (10, 11), I will in the following present some key methodological limitations along with recommendations to improve clinical research, particularly individual, parallel-group, randomized clinical trial methodology, within the field.

## Balancing beneficial and harmful effects

“First do no harm” is an important injunction in all medical interventions (12). It implies that both beneficial *and* harmful effects of any intervention should always be assessed. While beneficial effects (e.g., symptom reduction and quality of life) have been thoroughly assessed in psychotherapy trials for BPD, the harmful effects of psychotherapy for BPD are currently unclear due to lack of data (5).

A universal definition of a harmful effect of psychotherapy currently does not exist, and therefore, the appropriate way of assessing harmful effects of psychotherapy can been discussed (13, 14). Relevant harmful effects for BPD patients could be self-harm, suicidality, and, more broadly, serious adverse events (15). There are many other types of harmful effects that have been reported, for example clinical deterioration or treatment non-response (14). The problem with outcomes such as response, non-response, or deterioration is that they are often based on a dichotomization of a continuous scale. For example, trialists often dichotomize the Hamilton Depression Rating Scale (HDRS) by transforming the overall HDRS score between 0 and 52 into a dichotomous score comparing responders (≥ 50% improvement) to non-responders (≤50% improvement) (16). However, several studies have shown that dichotomization of continuous data can bias results (17, 18). For example, a participant who improves by ≥ 50% is defined as a responder, whereas a participant who improves by 49% is defined as a non-responder, and thus the apparent difference becomes inflated (16).

A more objective way of assessing harmful effects is by applying the proportion of participants with one or more serious adverse events as defined by the International Conference on Harmonization of technical requirements—Good Clinical Practice (ICH-GCP) guidelines (19) as an outcome. According to ICH-GCP, a serious adverse event is defined as any adverse event that results in death, is life-threatening, requires hospitalization or prolongation of existing hospitalization, or results in persistent or significant disability or incapacity (19). When assessing serious adverse events with the ICH-GCP definition, two blinded investigators should independently go through patient medical records and record all events according to these criteria. An advantage of this method is that all events should be classified regardless of their etiology, and the investigators thus avoid having to differentiate a disease-specific event (e.g., a suicide or suicide-attempts) from a non-disease-related event (e.g., an accident) (20). When employing the ICH-GCP definition, a potential suicide or suicide-attempt will be included as an event, and can be categorized in a serious adverse events table. If the number of randomized participants is high enough, the non-disease-related serious adverse events will be equal in both groups, and the “true” difference between the assessed interventions on the disease-related events becomes isolated. The inclusion of serious adverse events and other methods to assess harmful effects should be improved in future trials.

## Minimizing systematic errors (bias)

Causal inferences from randomized clinical trials can be undermined by errors in the design, conduct, analyses, and reporting leading to skewed estimates of the true intervention effects (bias) (21). Meta-epidemiological studies have shown that biased trial results typically overestimate beneficial effects and underestimate harmful effects of the experimental intervention (21, 22). Therefore, trials with high risk of bias may ultimately mislead clinical decisions.

In the Cochrane review of psychotherapy for BPD, all trials were assessed as at unclear or high risk of bias in a least one domain (5). The most biased domains were blinding of outcome assessors, incomplete outcome data, selective outcome reporting, and other bias (covering what the authors refer to as: attention, affiliation, or adherence bias, all of which can be understood as a bias arising from the trialists allegiance to one of the assessed interventions) (5). The authors did not assess bias associated with blinding of participants or clinicians. However, blinding of all key persons involved in a trial should be implemented whenever possible (23). Some key persons are more easily blinded than others, e.g., outcome assessors, data managers, statisticians, the data safety and monitoring committee, and the decision makers (23), and the blinding status of these persons could easily be implemented in future trials. Whether participants and clinicians could (and should) be blinded can be discussed. There are obviously practical challenges associated with delivering a treatment without being aware of its theoretical foundation. Furthermore, there are clinical challenges as self-confirming response expectancies associated with lack of blinding can be considered an “active ingredient” of e.g., cognitive behavioral therapy (23). On the other hand, lack of blinding of participants and clinicians can be understood as a bias in line with other unblinded persons involved in a trial. For a more detailed discussion about this dilemma, please consult Juul et al. (23).

Furthermore, I want to specifically highlight the problem with incomplete outcome data bias in BPD trials. Participants with BPD, and particularly adolescent populations, can be difficult to engage in follow-up interviews, once they are enrolled in a trial. If the degree of missing data is significant, this can seriously threaten the validity of the trial results. In systematic reviews, we generally tend to assess trials at low risk of incomplete outcome data bias, if missing data is less than 5% of all randomized participants (24). The 5% cutoff is not definitive, but it is a coarse rule of thumb. When comparing this rule with the missing data values sometimes exceeding 33% in psychotherapy trials with BPD (25), we as trialists must consider very carefully, how to better design trials at lower risks of incomplete outcome data bias. Multiple imputation, and other methods to statistically account for missing data can be used, if missing data is impossible to ignore. However, if the proportions of missing data are very large (for example, more than 40%) on important outcomes, no statistical method can solve that problem, and the trial results may then be considered hypothesis-generating only (24).

Usually, missing data will affect outcomes that require an action from the participant, e.g., if they need to fill out questionnaires or participate in a clinical interview. One solution could be to minimize the use of such outcomes to make participating in a trial less overwhelming for patients, while also considering more objective outcomes that can be retrieved from, e.g., national patient registries or medical records by (blinded) research personnel; i.e., hospitalization, suicide-related behavior, and employment status.

## Standardization of PICOs

In the Cochrane review of psychotherapy for BPD, most trials included small sample sizes, with the number of participants ranging from 7 to 151 (5). Furthermore, heterogeneity was observed in the selection of interventions and outcomes, hindering the pooling of effect estimates. The small sample sizes and the inability to pool results led to imprecise effect estimates (5). Therefore, future BPD trials should preferably be larger, and aim to assess similar PICOs (participants, interventions, comparators, and outcomes), so that pooling trials in a systematic review becomes less influenced by heterogeneity.

### Participants

The need to replicate existing trials is an aim that pragmatically conflicts with the wish for more personalized psychotherapy. BPD is a heterogeneous disorder with many potential subgroups (26). The more subgroups that are identified (e.g., patients with different levels of symptom severity at baseline), the more participants are needed, either if we should start multiple trials for every subgroup, or perform subgroup analyses embedded within a trial. While we should aim to conduct trials with different types of BPD patients to cover the whole spectrum of patient characteristics, we should still be mindful of which conclusions can be drawn from subgroup analyses that are exploratory by nature (8).

### Interventions and comparators

Several standardized interventions specifically developed to treat BPD and BPD-related symptoms have already been developed, including DBT, MBT, schema therapy (ST), and transference focused psychotherapy (TFP) (5). While developing new and potentially improved interventions may seem promising, more trials assessing the effects of the already existing BPD-interventions are still needed, if we want to confirm or reject intervention effects on several important outcomes (5). Furthermore, an adequate description of the assessed trial interventions and comparators is required for trialists to design replication trials and for clinicians to reliably implement interventions (27, 28). Both the experimental and the control interventions need to be described in detail (29). To improve reporting of interventions, the Template for Intervention Description and Replication checklist and guide (TIDieR) has been developed (28), which can be used as an addition to the CONSORT guideline for reporting of trials (30).

### Outcomes

Existing BPD trials have used a wide range of outcome measures, which makes it difficult to synthesize data in systematic reviews (31). Furthermore, the selected outcomes do not always adequately reflect BPD patient experience (31). This calls for a discussion of which outcomes are the most patient-important. A standard set of patient-reported outcomes for the International Classification of Diseases—11th version (ICD-11) personality disorder classification has recently been proposed by the International Consortium for Health Outcomes Measurement (ICHOM) multidisciplinary working group (32). However, these recommendations did not cover more objective outcomes. Agreeing on a core outcome set (COS) for future trials of psychotherapy for BPD is needed and will improve the development of evidence-based treatment guidelines in the future (31). In my opinion, the field could move forward by including both continuous outcomes like quality of life and symptom severity, and also dichotomous outcomes like hospitalization, self-harm, suicide or suicide-attempts and employment status. However, the development of a COS is highly needed and should involve key stakeholders including researchers and methodologists but also patient organizations, relatives, clinicians, funders, and administrators, and should follow strict development guidelines provided by the Core Outcome Measures in Effectiveness Trials (COMET) initiative (33).

## Improving conflict of interest disclosures and retrieve unpublished data

Psychotherapy research has long struggled with the potential bias of trialists who believe in the superiority of one psychological intervention over another, a phenomenon typically referred to as attention bias or researcher allegiance (34). Researcher allegiance is a heterogeneous construct ranging from developing the treatment manual to advocating for it to contributing to a related disease model to, ultimately, conducting a trial showing results in favor of the new experimental intervention (35). Examples of financial conflicts of interest in psychotherapy trials are when trialists also have financial gains from e.g., professional trainings of that particular intervention, books, therapy manuals, courses, speaker’s fees, paid advisory positions, grants etc. (34).

One way of assessing the potential impact of conflicts of interest is for systematic reviewers of randomized clinical trials to carefully look for signs of publication bias when performing a meta-analysis. Publication bias refers to the publication or non-publication of research findings, depending on the direction of the results (36). Trialists with a strong allegiance to an experimental intervention may decide not to publish the trial results, if the results do not comply with their expectations. Pre-registration of trials at registries such as www.clinicaltrials.gov are now required when launching a trial. Pre-registration of trials minimizes study publication bias (when trials showing negative or no effect are not published) and selective outcome reporting bias (when trialists fail to report unfavorable data, include only a subset of data analyzed, or change or omit the outcome of interest in the interest of statistical significance) (37). However, while pre-registration of trials are a methodological safeguard, both publication bias and selective outcome reporting bias may still occur.

Assessment of publication bias can be performed by visually inspecting funnel plots (36) and by statistically testing the funnel plot asymmetry using various tests depending on the outcome of interest (36). A funnel plot is a scatter plot of the effect estimates from individual trials against some measure of each trial’s size or precision (usually the standard error) (38).

In the Cochrane review of psychotherapies for BPD, the inspection of the funnel plot suggested potential bias (small asymmetry) (5). Furthermore, the authors assessed almost a third of the included trials as being influenced by “other bias” such as attention, affiliation, or adherence bias, indicating a potential conflict of interest in most included trials.

To control for researcher allegiance on a trial level, trialists should aim to implement blinding of all possible key persons involved in data collection, analysis, interpretation, and dissemination of the trial results (23). Furthermore, disclosures of financial conflicts of interest for all contributing authors of published trial reports should be improved (34), and, on a systematic review level, authors should carefully try to retrieve unpublished data. Moreover, all trials need to be transparently registered before launch and all trial data transparently registered after analysis and publication (39).

## Conclusion

While we have already come a long way in both designing and implementing structured, manualized psychotherapies for BPD, and in assessing their effects in randomized clinical trials, the current evidence is still restricted by substantial methodological limitations. In this paper, I have highlighted some key methodological limitations and suggested the following recommendations: *balancing beneficial and harmful effects, minimizing systematic errors (bias), standardization of PICOs, improving conflict of interest disclosures, and retrieving unpublished data.* Improving these methodological limitations can lead to us potentially identifying more evidence-based treatments, which may ultimately result in better care for BPD patients.

The reader should take into account the potential limitations of this paper. The recommendations are based on my own opinion, rather than originating from an international consortium of experts. Thus, the recommendations are in no way exhaustive, but may serve as a stepping stone for further improvement in the field.

Evidence-based medicine is the conscientious, explicit, and judicious use of best evidence in making decisions about the care of individual patients (6). Evidently, clinicians should, by default, offer psychotherapy supported by the best available evidence. It is our job as both clinicians and trialists to continuously make that evidence as trustworthy as possible.

## Author contributions

The author confirms being the sole contributor of this work and has approved it for publication.
